# Aversive Pavlovian inhibition in adult attention-deficit/hyperactivity disorder and its restoration by mindfulness-based cognitive therapy

**DOI:** 10.3389/fnbeh.2022.938082

**Published:** 2022-07-25

**Authors:** Dirk E. M. Geurts, Hanneke E. M. den Ouden, Lotte Janssen, Jennifer C. Swart, Monja I. Froböse, Roshan Cools, Anne E. M. Speckens

**Affiliations:** ^1^Donders Institute for Brain, Cognition and Behaviour, Radboud University, Nijmegen, Netherlands; ^2^Department of Psychiatry, Radboud University Medical Centre, Nijmegen, Netherlands; ^3^Institute of Experimental Psychology, Heinrich Heine University of Düsseldorf, Düsseldorf, Germany

**Keywords:** ADHD, Pavlovian to instrumental transfer, mindfulness based cognitive therapy, inhibition, impulsivity

## Abstract

**Background:**

Control over the tendency to make or withhold responses guided by contextual Pavlovian information plays a key role in understanding impulsivity and hyperactivity. Here we set out to assess (1) the understudied relation between contextual Pavlovian inhibitory control and hyperactivity/impulsivity in adults with ADHD and (2) whether this inhibition can be enhanced by mindfulness based cognitive therapy (MBCT).

**Methods:**

Within the framework of a randomized controlled trial 50 Adult ADHD patients were assessed before and after 8 weeks of treatment as usual (TAU) with (*n* = 24) or without (*n* = 26) MBCT. We employed a well-established behavioral Pavlovian-to-instrumental transfer task that quantifies Pavlovian inhibitory control over instrumental behavior.

**Results:**

Task results revealed (1) less aversive Pavlovian inhibition in ADHD patients with clinically relevant hyperactivity/impulsivity than in those without; and (2) enhanced Pavlovian inhibition across all ADHD patients after TAU+MBCT compared with TAU.

**Conclusion:**

These findings offer new insights in the neurocognitive mechanisms of hyperactivity/impulsivity in ADHD and its treatment: We reveal a role for Pavlovian inhibitory mechanisms in understanding hyperactive/impulsive behaviors in ADHD and point toward MBCT as an intervention that might influence these mechanisms.

## Introduction

Individuals diagnosed with attention-deficit/hyperactivity disorder (ADHD) have difficulties with controlling their behavior appropriately with respect to environmental demands. Two key cognitive systems that control our behavior with respect to the environment are the Pavlovian and instrumental systems (Dickinson and Balleine, 2002; Dolan and Dayan, 2013). Especially problems in Pavlovian control of goal-oriented instrumental behaviors are associated with a wide variety of psychiatric problems (e.g., Dayan et al., 2006; Heinz et al., 2016; Hallquist et al., 2018). This form of behavioral control might be key to adaptive inhibitory control which has since long been proposed to be central to understanding problems in ADHD (Barkley, 1997). Moreover, aberrant Pavlovian control over instrumental behavior can lead to maladaptive impulsivity in animals as well as in humans (Breland and Breland, 1961; Guitart-Masip et al., 2014; Hinojosa-Aguayo and González, 2020). This form of control has been shown to depend on monoaminergic transmission relevant for understanding ADHD (Dalley and Roiser, 2012; Geurts et al., 2013b; Salamone et al., 2015) and can specifically be modulated by methylphenidate (Swart et al., 2017). Nevertheless, it has received relatively little attention in human and animal ADHD research (Natsheh and Shiflett, 2015). To fill this gap in the literature, we tested whether Pavlovian control of instrumental behavior [i.e., Pavlovian to instrumental transfer (PIT)] is related to clinically relevant impulsivity/hyperactivity in ADHD. Therefore, we first compared Pavlovian control in adult ADHD patients diagnosed with and without clinically relevant impulsivity/hyperactivity. Second, we assessed the hypothesis that a mindfulness-based cognitive therapy (MBCT), i.e., an 8-week training program theoretically related to amending automatic tendencies (Segal et al., 2002) and shown to improve impulsivity/hyperactivity in ADHD (Janssen et al., 2018), should, accordingly, also modulate Pavlovian inhibitory control.

A wide range of animals, including humans, are endowed with mechanisms shaped throughout evolution that drive behavior (Dolan and Dayan, 2013). These drivers take advantage of environmental information carried by stimuli that predict motivationally salient future events or outcomes. The instrumental control system enables us to use specific actions when confronted with a certain stimulus to obtain a specific outcome (i.e., stimulus-action-outcome learning or operant conditioning). This system allows us to optimize our chances to achieve specific goals by learning when to exert specific actions and when not to act. Complementary to this instrumental control system, the Pavlovian control system regulates automatic, motivational responses in reaction to external and internal stimuli (Dolan and Dayan, 2013). This system enables us to associate neutral stimuli with motivationally salient outcomes in the environment (i.e., stimulus-outcome learning or classical conditioning). These neutral stimuli acquire part of the motivational properties of the outcome they are associated with (i.e., predict). When encountering these previously neutral, but now conditioned, stimuli (CS) again, the automatic preparatory reaction to the outcome will be elicited in response to these Pavlovian CSs. Critically, it has long been recognized that these two behavioral control systems do not act in separation, but interact. Pavlovian CS can (de) motivate ongoing instrumental behavior based on the valence (appetitive or aversive) of the Pavlovian CS (Rescorla and Solomon, 1967): Pavlovian CS that predict punishment (i.e., aversive Pavlovian CS) have the tendency to inhibit, whereas Pavlovian CS that predict reward (i.e., appetitive Palvovian CS) can activate instrumental behavior (Rescorla and Solomon, 1967; Huys et al., 2011; Geurts et al., 2013a). These interactions between instrumental and Pavlovian control of behavior are thought to be shaped by evolution and have adaptive properties in terms timing actions (i.e., when to make, and when not to make an action) to optimize gaining rewards and avoiding punishment at relatively low computational cost (Dolan and Dayan, 2013). However, too much or too little influence of the Pavlovian system on instrumental behavior has been proposed as a driver of several maladaptive behaviors (e.g., Dayan et al., 2006; Heinz et al., 2016; Hallquist et al., 2018). Too much potentiation of instrumental behavior by appetitive cues, or too little inhibition by aversive cues is linked to impulsive behavior in real life (Watson et al., 2014; Garbusow et al., 2016; Heinz et al., 2016; Hallquist et al., 2018). This latter source of disinhibition, i.e., disinhibition in the face of aversive affect, has been widely recognized to play a role in externalizing psychopathology, mainly under the umbrella of negative urgency (Whiteside and Lynam, 2001). Negative urgency has recently indeed been related to Pavlovian control of instrumental behavior in healthy controls (Hinojosa-Aguayo and González, 2020). However, whether the impact of appetitive activating and aversive inhibitory processes on instrumental behavior contributes to impulsivity/hyperactivity in ADHD remains an open question. We will test this specific hypothesis in ADHD patients diagnosed with and without clinically relevant impulsivity/hyperactivity symptomatology. Specifically, we compare patients diagnosed with the DSM-IV combined or hyperactive/impulsive subtype (both including relevant impulsive-hyperactive symptomatology) with those with the primarily inattentive subtype (without diagnosed impulsivity/hyperactivity).

The hypothesis that both increased appetitive PIT and decreased aversive PIT might drive impulsivity in ADHD can only be tested causally through an intervention study. A key prediction is that effective treatment of ADHD should modulate the effect of Pavlovian cues on instrumental behavior. One candidate for this is MBCT. MBCT has significant beneficial effects in ADHD (Cairncross and Miller, 2016; Gu et al., 2017; Hepark et al., 2017; Janssen et al., 2018), as well as impulsivity symptoms trans-diagnostically (Franco et al., 2016). It is a highly protocolled intervention that changes how patients deal with thoughts, emotions, bodily feelings and urges in reaction to both external and internal stimuli. Patients become more aware of internal and external triggers and consequent automatic patterns such as avoidance of aversive stimuli or attachment to appetitive stimuli, and learn to (initially) disengage from automatic reactivity (Segal et al., 2002, p. 217). Indeed, MBCT has been shown to reduce impulsivity/hyperactivity (Gu et al., 2017; Hepark et al., 2017; Janssen et al., 2018), improve self-reported adaptive inhibition (Hepark et al., 2017; Janssen et al., 2018) and increases experimentally measured behavioral inhibition (see for meta-analyses and critical notes; Lao et al., 2016; Vago et al., 2019). Moreover, previous findings from our group suggest that effects of MBCT on self-reported adaptive inhibition mediated the effects of MBCT on clinician rated ADHD symptoms (Geurts et al., 2020). Taken together, to test the hypothesis that aberrant PIT may drive impulsive responding in ADHD, we will assess whether MBCT changes the inhibitory or activating effects of Pavlovian cues. In line with the hypothesized relation between impulsivity and Pavlovian control, we expect MBCT to diminish the motivating effect of appetitive Pavlovian CS and to enhance the inhibiting effect of aversive Pavlovian CS on instrumental behavior, leading to more inhibition and less impulsivity/hyperactivity, respectively.

## Materials and methods

### Trial design and procedure

This behavioral intervention study was embedded in a multi-center randomized controlled trial (RCT) investigating the impact of MBCT in addition to TAU on adults with ADHD (NCT02463396) (Janssen et al., 2015, 2018). For this trial, a total of 120 adults with ADHD according to the criteria of Diagnostic and Statistical Manual of Mental Disorders—4th edition (DSM-IV-TR) (American Psychiatric Association, 2000) were randomized to either MBCT in addition to treatment as usual (MBCT + TAU) or TAU only. The eligibility criteria, study procedure and CONSORT diagram are described fully in the protocol paper (Janssen et al., 2015) and the main treatment outcome paper (Janssen et al., 2018) of the overarching RCT. Clinical outcome measures were assessed before (T0) and directly after (T1) and 3 months (T2) after MBCT or TAU. Behavioral data on the PIT task were collected before (T0) and after MBCT or TAU (T1). On each of these two test-days, patients were seated in front of a laptop and conducted the PIT computer task.

### Patients

For the current study, behavioral data on the PIT task were collected from a subset of patients assessed at one site (RadboudUMC): 68 patients were asked to participate. One patient declined, which resulted in 67 patients participating in the pre-intervention test session. On the post-intervention test session 60 (90%) patients participated and 7 declined to participate. Unfortunately, there was a loss of 10 data sets on pre-intervention due to a technical (back-up) error, leaving 50 full data sets (MBCT+TAU: *n* = 24; TAU only *n* = 26) to be analyzed (for demographics see Table 1; see Supplementary Data for flow-chart of inclusions).

**TABLE 1:** Baseline demographic and clinical characteristics.

	MBCT+TAU (*n* = 24)		TAU (*n* = 26)		P (Phi/T statistics)
**Demographic characteristics**					
Female gender	13	54.2%	16	61.5%	0.28 (Phi = –0.075)
Age; *M* (*SD*)	42.6	12.4	39.0	10.5	0.26 (T_48_ = –1.1)
**Clinical characteristics**					
Subtype of ADHD, DSM-IV					
Inattentive type	13	54.2%	16	61.5%	0.87 (Phi = –0.28)
Hyperactive/impulsive type	0	0%	0	0%	
Combined type	10	41.7%	9	34.6%	
Not otherwise specified type	1	4.2%	1	3.8%	
ADHD symptoms (CAARS-INV)					
Subscales:					
Inattention	16.9	5.2	18.6	3.8	0.20 (T_48_ = 1.3)
Hyperactive/impulsive	11.7	6.5	14.2	5.9	0.15 (T_48_ = 1.5)
Total	28.6	9.4	32.8	8.4	0.10 (T_48_ = 1.7)
Use of ADHD medication	17	70.8%	14	53.8%	0.22 (Phi = 1.53)

### Intervention

#### Mindfulness-based cognitive therapy

MBCT (Segal et al., 2002) is an 8-week group-based intervention of 2.5 h each, plus a 6-h silent day between session 6 and 7. In short, the program included mindfulness practice (bodyscan, gentle yoga, sitting and walking meditation) combined with daily life practices, psycho-education, Cognitive Behavioral Therapy (CBT) techniques, group discussions, and inquiry into present moment experiences. By this procedure patients are taught to become more aware of dysfunctional automatic patterns, such as avoidance of aversive stimuli or grasping of appetitive stimuli and to consciously disengage from these patterns (Segal et al., 2002, for further detail see the protocol paper; Janssen et al., 2015).

#### Treatment as usual

TAU reflected the usual treatments of ADHD patients in various mental health centers across the Netherlands, consisting of pharmacotherapy and psychosocial treatment, such as psycho-education and cognitive behavioral therapy.

### Assessments

#### Clinical assessments

Clinical assessments are presented in Table 1. The primary outcome measure was total ADHD symptoms according to the 30-item Conners’ Adult ADHD Rating Scale (Conners et al., 1999), scored by a blinded clinician (CAARS-INV). In line with the findings of the overarching RCT (Janssen et al., 2018, *n* = 120), MBCT significantly reduced ADHD symptoms in our subsample of the total patient group with a moderate effect size (Treatment (MBCT/TAU) × Day (pre/post) interaction on CAARS-IVNV ADHD score: *F*(1, 48) = 5.2, *p* = 0.028; independent sample *t*-test pre MBCT: *t*(48) = 1.7, *p* = 0.098; post MBCT: *t*(48) = 2.8, *p* = 0.007; paired sample *t*-test (pre vs. post): MBCT+TAU: *t*(23) = 4.4, *p* < 0.001; TAU: *t*(25) = 3.7, *p* = 0.007).

Overall functioning was measured using the 45-item Outcome Questionnaire 45 (OQ-45), which offers a comprehensive review of overall life functioning (Lambert et al., 1996). Items are scored on a five-point rating scale, ranging from never (0) to almost always (4), with a maximum score of 180 points (a higher score means worse overall functioning) (DE Jong et al., 2007).

#### Pavlovian to instrumental transfer task

**FIGURE 1:**
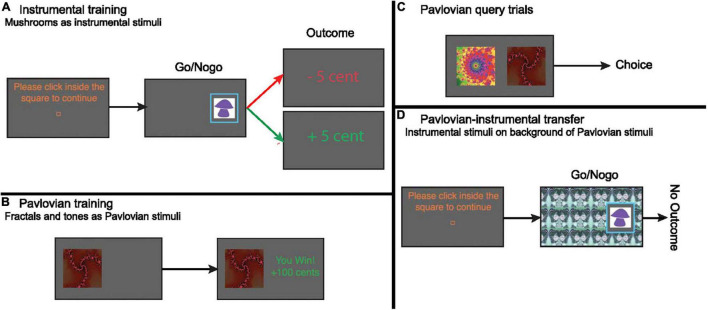
**(A)** Instrumental conditioning. To center the cursor, participants clicked in a central square. Participants needed to choose whether to move the cursor toward the mushroom and click inside the blue frame onto the mushroom (go), or do nothing (NoGo). Outcomes were presented immediately after go actions, or after 1.5 s (i.e., NoGo). There were 3 “good” (go) and 3 “bad” (NoGo) instrumental stimuli. Collecting a “good” (correct go) and not collecting a “bad” (correct NoGo) stimulus was rewarded most of the time (75% veridical outcome). Vice versa, collecting a “bad” (incorrect go) and not collecting a “good” (incorrect NoGo) stimulus was punished most of the time (75% veridical outcome). There were 60 trials in total. Instrumental stimuli were different for both days. **(B)** Pavlovian conditioning. Participants passively viewed stimuli and heard auditory tones, followed by wins (+10/+100), losses (–10/–100), or neutral outcomes (0). There were five fractal/tone combinations. Each combination was displayed 12 times. **(C)** On Pavlovian query trials, participants chose between two Pavlovian stimuli. Query trials were administered after every five Pavlovian conditioning trials. **(D)** Pavlovian-instrumental transfer. Participants responded to the instrumental stimuli trained during the instrumental conditioning stage, with Pavlovian stimuli tiling the background. No outcomes were presented, but participants were instructed that their choices counted toward the final total. No explicit instructions about the contribution of Pavlovian stimuli toward the final total were given. During this phase we assessed the impact of the Pavlovian CSs on instrumental choice (go/NoGo).

We used a PIT task that allowed us to assess the influence of appetitive and aversive Pavlovian CS on instrumental approach actions. This task was identical to the approach blocks used in Huys et al. (2011, 2016) and Geurts et al. (2013b). In short, the task consisted of an instrumental conditioning, a Pavlovian conditioning and a PIT stage (Figure 1).

##### Instrumental conditioning

The instrumental task (Figure 1A) was a go/NoGo task, framed in terms of collecting “good” and “bad” mushrooms. Patients chose whether to collect the mushroom by moving the mouse toward and clicking on the mushroom (go) within a response-window of 1.5 s, or not collect the mushroom by abstaining from a response for 1.5 s (NoGo). The outcome (±5 cents) was then presented in the middle of the screen. Reinforcements were probabilistic, with the “correct” response for each mushroom leading to reward on 75% of the trials and to punishment otherwise. For the “incorrect” response these probabilities were reversed. Correct trials were those on which they collected a “good” mushroom or refrained from collecting a “bad” mushroom. Patients thus had to learn the better response for each stimulus from the probabilistic, noisy reinforcement feedback. There were 3 “good” (go) and 3 “bad” (NoGo) mushrooms, meaning that the possible actions (i.e., collect or not collect) could be followed by both rewards and punishments.

Analyses and results on the instrumental conditioning stage are reported in Supplementary Material.

##### Pavlovian conditioning

The second part of the task consisted of a separate Pavlovian conditioning procedure. Five compound Pavlovian CS, consisting of a fractal visual stimulus (Figure 1B) and a tone, were deterministically paired with outcomes. The appetitive (S*^P^*_++_, S*^P^*_+_) and aversive (S*^P^*_–_, S*^P^*_––_) Pavlovian CSs predicted a gain/loss of 100 or 10 cents, respectively, while the neutral CS (S*^P^*_0_) was followed by an outcome of 0 cent. To ensure that patients paid attention, a query trial was presented on every fifth trial. Patients then had to choose between two different Pavlovian CS (Figure 1C) without any reinforcement. In addition, we asked patients to rate how much they liked the presented CS before and after the experiment on a visual analog scale (VAS).

Analyses and results on the Pavlovian conditioning stage are reported in Supplementary Material.

##### Pavlovian-instrumental transfer phase

This was the main phase of interest. Patients needed to choose whether to collect (go) or not collect (NoGo) the same mushrooms as in the instrumental training phase, while the Pavlovian CS now tiled the entire background (Figure 1D). No outcomes were presented during this phase to exclude further instrumental conditioning. Patients were instructed to continue performing the instrumental task; that choices were still earning them the same outcomes and were being counted; but that they would not be told about the outcomes during this phase. Thus, in this phase, we could assess the impact of the Pavlovian CS on the previously learned instrumental go/NoGo choices.

### Data analysis

The primary effect of interest was the activating and inhibiting impact of the appetitive and aversive Pavlovian CSs on instrumental go/NoGo choices, respectively.

First, we assessed the relation between clinical impulsivity-hyperactivity and PIT: In the introduction we introduced two possible links between hyperactivity-impulsivity on the one hand and appetitive and aversive PIT on the other: Impulsivity-hyperactivity could theoretically be instantiated differentially by (i) exaggerated appetitive PIT, i.e., too much instrumental potentiation in the face of an appetitive Pavlovian CS and (ii) diminished aversive PIT, i.e., too little inhibition in the face of an aversive Pavlovian CS (Watson et al., 2014; Garbusow et al., 2016; Huys et al., 2016; Hallquist et al., 2018). We assessed these differential associations at baseline by assessing differences in PIT between the combined subtype (including hyperactivity-impulsivity) and the inattentive subtype (not including hyperactivity/impulsivity) of ADHD. Thus, we employed two generalized linear mixed effects models (GLMM) with, respectively, Pavlovian CS Appetitive (S*^P^*_++_ /S*^P^*_*n*_) and Aversive (S*^P^*_*n*_/S*^P^*_––_ as within subject factor and ADHD subtype (combined/inattentive) as between-subject factor.

Second, to test whether MBCT modulated appetitive and aversive PIT we used a GLMM including the within-subject factors Pavlovian CS Valence (5 levels: S*^P^*_++_/S*^p^*_+_/S*^P^*_*n*_/S*^P^*_–_/S*^P^*_––_) and Day (Pre vs. Post treatment), and the between-subject factors Treatment Group (TAU+MBCT vs. TAU).

We used GLMMs to account for both between- and within-subject variability. We used the lme4 package in R (Bates et al., 2015; R Development Core Team, 2015). All GLMMs included all main effects and interactions as well as a full random effects structure to reduce inflation of Type I error (Barr et al., 2013).

Furthermore, to interpret the results of the above analyses as true changes in the interaction between Pavlovian and instrumental control, i.e., PIT, there should be no differences between the Treatment groups in task performance during the instrumental and Pavlovian training *per se* on Day 1 or a difference in change between the Groups from pre- to post treatment in these parts of the training. We assessed whether this was the case by using, where appropriate, *t*-tests and repeated measure ANOVA’s with, respectively, average performance at the end of the instrumental training stage (mean correct after more than 5 stimulus presentations), average performance at the end of Pavlovian training (mean correct after more than 5 query trials) and VAS ratings from pre-to post Pavlovian conditioning as dependent variables.

We note, that we did not pursue analysis of reaction time, because previous reports (Huys et al., 2011; Geurts et al., 2013b) with this paradigm did not find any meaningful effects on this outcome measure.

## Results

### General Pavlovian to instrumental transfer effects

Across Treatment Group and Day we replicated the expected PIT effect: appetitive Pavlovian CS activated (i.e., appetitive PIT), whereas aversive Pavlovian CS inhibited (i.e., aversive PIT) instrumental approach actions [main effect of Pavlovian CS Valence: _X_^2^ = 17.4, *p* = 0.002; simple contrast appetitive PIT (S*^P^*_*n*_/ S*^P^*_++_): _X_^2^ = 4.9, *p* = 0.026); simple contrast aversive PIT (S*^P^*_*n*_/S*^P^*_–_): _*FRS*:X_^2^ = 7.4, *p* = 0.006].

### Aversive Pavlovian inhibition is related to clinical impulsivity-hyperactivity

**FIGURE 2:**
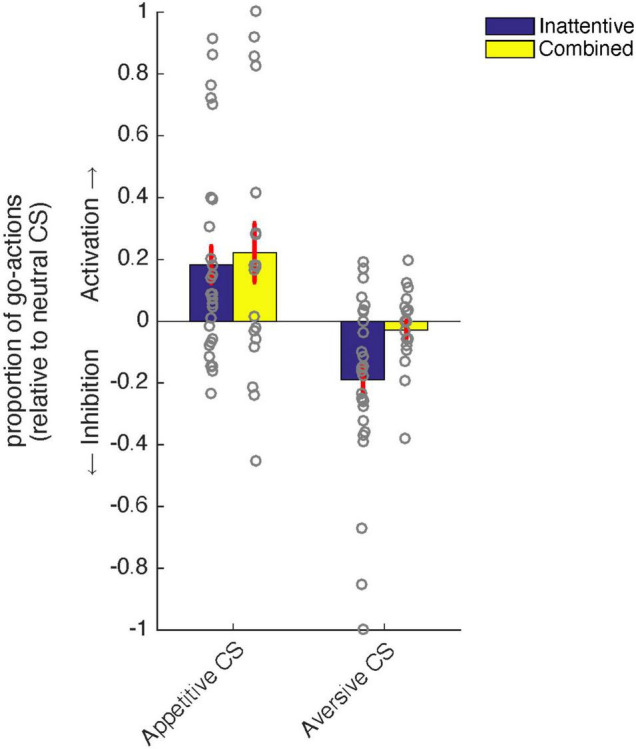
Relation between ADHD subtype [combined (yellow) vs. inattentive (blue)] and PIT. Patients with the inattentive subtype showed significant aversive inhibition of instrumental behavior in the context of an aversive Pavlovian conditioned stimulus (CS), while this was not the case for patients diagnosed with the combined subtype. There were no differences between ADHD subtypes in terms of appetitive activation of instrumental behavior. Error bars represent standard errors of the mean.

Specific analyses, targeted at clinically diagnosed impulsivity/hyperactivity and its relation to aversive and appetitive PIT, respectively (see section “Introduction” and “Materials and methods”), revealed that aversive PIT was absent for those patients diagnosed with ADHD including impulsivity/hyperactivity (i.e., the combined subtype) compared with patients with ADHD with primarily inattentive symptoms [Figure 2, Subtype (combined/inattentive) × Pavlovian CS Valence (S*^P^*_*n*_/ S*^P^*_––_):_X_^2^ = 4.6, *p* < 0.031, Table 2]. More specifically, behavioral inhibition by aversive Pavlovian CS was not significant in patients diagnosed with the combined subtype (_X_^2^ = 1.5, *p* = 0.22) and significant for the inattentive subtype (_X_^2^ = 12.77, *p* < 0.001). No such effects were found for appetitive PIT [Subtype (combined/inattentive) × Pavlovian CS Valence (S*^P^*_*n*_/ S*^P^*_++_): _*FRS*:X_^2^ = 0.1, *p* = 0.73].

**TABLE 2:** Baseline demographic and clinical characteristics compared between those patients diagnosed with subtypes with and without hyperactivity/impulsivity.

	Combined subtype including hyperactivity/impulsivity		Inattentive subtype		*p*
**Demographic characteristics**					
Female gender	19/29	65.5%	9/19	47.4%	0.21
Age; M(SD)	38.1	(9.9)	44.4	(12.7)	0.060
**Clinical characteristics**					
ADHD symptoms (CAARS-INV)					
Total score	33.6	(8.3)	26.9	(9.3)	0.012
Inattention subscale	18.2	(5.0)	17.4	(4.2)	0.6
Hyperactive/impulsive subscale	15.4	(4.8)	9.5	(6.6)	<0.001
Outcome questionnaire	55.7	(15.6)	63.7	(18.9)	0.12
Use of ADHD medication	17/29	(58.6%)	12/19	(63.2%)	0.8

### Mindfulness based cognitive therapy increased aversive Pavlovian inhibition over instrumental behavior

**FIGURE 3:**
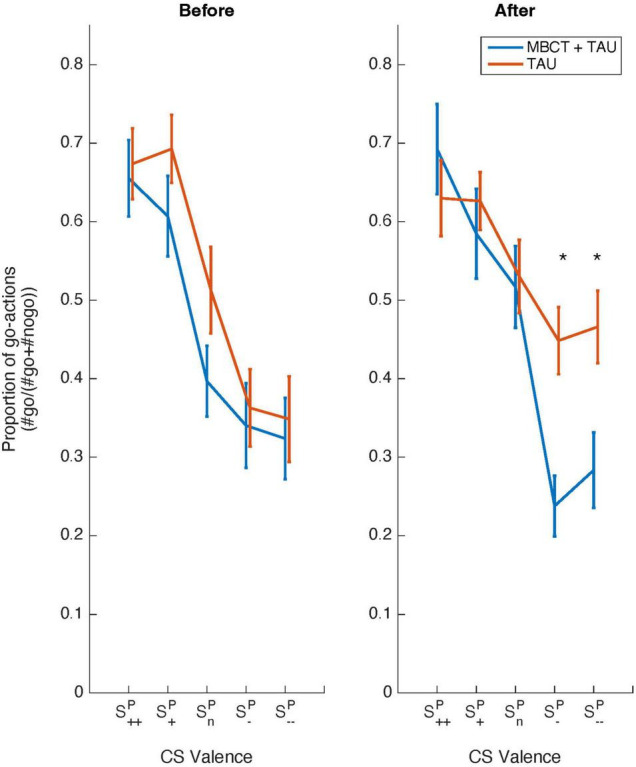
Behavioral data from the Pavlovian-instrumental transfer stage as a function of Treatment. Shown are choice data (proportion of go-actions) as a function of Pavlovian CS Valence (S*^P^*_++_/S*^P^*_+_/S*^P^*_*n*_/S*^P^*_–_/S*^P^*_––_) and Day (before vs. after) for a group receiving mindfulness based cognitive therapy and treatment as usual (MBCT+TAU, blue line) and a group receiving treatment as usual (TAU) only (red line). The group receiving MBCT shows increased aversive inhibition after MBCT (*p* < 0.05) compared to the TAU only group. Error bars represent standard errors of the mean.

Notably, we found that MBCT modulated PIT as is revealed by a Treatment Group × Day × Pavlovian CS Valence interaction (_X_^2^ = 12.9, *p* = 0.011, Figure 3). Simple contrast analyses showed that this interaction was driven by changes in aversive PIT [Treatment Group × Day × Pavlovian CS Valence (S*^P^*_*n*_/ S*^P^*_––_): _X_^2^ = 7.4, *p* = 0.006] and not appetitive PIT [Treatment Group × Day × Pavlovian CS Valence (S*^P^*_*n*_/ S*^P^*_++_): _X_^2^< 0.1, *p* = 0.86]. Indeed, as revealed by the pattern in Figure 3, aversive PIT was enhanced post MBCT [Day (pre vs. post) × Pavlovian CS Valence (S*^P^*_*n*_/ S*^P^*_––_): _X_^2^ = 5.6, *p* = 0.018], but not post -TAU [Day (pre vs. post) × Pavlovian CS Valence (S*^P^*_*n*_/ S*^P^*_––_): _X_^2^ = 3.3, *p* = 0.069]. Moreover, there was no difference in PIT at baseline between the groups (Pre: Treatment Group × Pavlovian CS Valence: _X_^2^ = 5.3, *p* = 0.26), but there was a difference after MBCT/TAU (Post: Treatment Group × Pavlovian CS Valence: _X_^2^ = 11.0, *p* = 0.027), which was driven by enhanced aversive PIT for the MBCT compared to the TAU group [Post: Treatment Group × Pavlovian CS Valence (S*^P^*_*n*_/ S*^P^*_––_): _X_^2^ = 5.1, *p* = 0.023].

Thus, MBCT increased the inhibitory effects of aversive Pavlovian CS on instrumental behavior and left unchanged the activating effect of appetitive Pavlovian CS.

### Instrumental and Pavlovian training

To interpret the above findings as true changes in the interaction between Pavlovian and instrumental control, i.e., PIT, there should be no (explanatory) differences between the Treatment groups at Day1 or a difference in change from pre- to post treatment between the groups in task performance at the end of instrumental and Pavlovian training. Indeed, we did not find evidence for such differences. Instrumental conditioning was successful as revealed by an above chance performance across the group at the end of the instrumental training on both days (one-sample *t*-test on mean correct choices after > 5 presentations vs. chance level (0.5 correct): Day1: *t*_(49)_ = 4.2, *p* < 0.001: 0.59, 95% CI 0.54–0.63; Day2: *t*_(49) _ = 4.6, *p* < 0.001; mean correct choices after > 5 presentations: 0.61, 95% CI 0.57–0.67). Moreover, performance did not differ between Treatment at Day 1 (two sample *t*-test on mean correct choices after > 5 presentations): *t*_(48)_ = 0.8, *p* = 0.86, 95% CI of difference: –0.08 to 0.09) nor was performance dependent on an interaction between Day and Treatment (_X_^2^< 0.1, *p* = 0.99). This was also the case for Pavlovian conditioning: Conditioning in terms of explicit associations between CS and outcomes resulted in above chance performance across Treatment group on both days (Day 1: one-sample *t*-test: mean = 0.88, 95% CI: 0.83–0.93, *t*_(49)_ = 14.7, *p* < 0.001: mean = 0.91, 95% CI: 0.87–0.96; Day2, *t*_(49)_ = 19.5, *p* < 0.001) and no group differences arose (Day 1: two sample *t*-test: *t*_(48)_ = –0.25, *p* = 0.80, 95% CI of difference: –0.12 to 0.09; interaction between Day and Treatment: _X_^2^ = 0.4, *p* = 0.53). Moreover, VAS liking ratings from before to after conditioning showed the expected pattern (appetitive stimuli were judged appetitive and aversive stimuli as aversive after training: Time (2 levels: pre/post conditioning) × Pavlovian CS Valence (5 levels: S*^P^*_++_/S*^p^*_+_/S*^P^*_*n*_/S*^P^*_–_/S*^P^*_––_) at Day 1: _X_^2^ = 29.4, *p* < 0.001) with again no difference in conditioning effects between the Treatment groups on Day1 [Group × Pavlovian CS Valence × Time (pre/post conditioning): _X_^2^ = 2.8, *p* = 0.093] or as a function of change from pre- to post treatment [Group × Day × Pavlovian CS Valence × Time (pre/post conditioning): _X_^2^< 0.1, *p* = 0.85].

## Discussion

Theory and data suggest that hyper(re)activity and impulsivity might be related to exaggerated appetitive Pavlovian activation and diminished aversive Pavlovian inhibition (Watson et al., 2014; Garbusow et al., 2016; Heinz et al., 2016; Hallquist et al., 2018). This prediction, however, remained untested for ADHD. We present two key findings. First, an ADHD diagnosis with clinically relevant impulsivity/hyperactivity was accompanied by an *absence* of aversive Pavlovian inhibition, while an ADHD diagnosis without clinically relevant impulsivity/hyperactivity was accompanied by the expected aversive Pavlovian inhibition, akin to multiple healthy control studies (e.g., Huys et al., 2011; Geurts et al., 2013a). In contrast to our expectations, we did not find a relation between appetitive Pavlovian activation and impulsivity/hyperactivity. Second, within a randomized controlled setting, MBCT enhanced this aversive Pavlovian inhibition across the whole group of patients.

Both our findings, the relation between impulsivity/hyperactivity and aversive Pavlovian inhibition and the strengthening of this inhibition through MBCT in ADHD, are particularly interesting when considering the wide ranging, adaptive effects of Pavlovian inhibitory processes in more detail. Pavlovian conditioned reactions have long been recognized to help the organism prepare (in a fast and computationally efficient manner) for the predicted outcome (Dickinson and Balleine, 2002). In case of appetitive outcomes these “preparations” increase the chances to benefit from this outcome. In the case of aversive outcomes, the Pavlovian behavioral reactions (e.g., inhibition) might prevent damage to the organism. Allowing predictors of aversive outcome (i.e., aversive Pavlovian CS) to influence behavior thus might instigate adaptive behavior. Moreover, aberrant Pavlovian mechanisms, e.g., too much appetitive attraction and/or too little aversive inhibition, are thought to play a role in psychiatric disorders such as major depressive disorder, different anxiety disorders, addiction (Huys et al., 2015; Heinz et al., 2016; Mkrtchian et al., 2017) and personality disorders associated with impulsive behaviors (Ly et al., 2016; Hallquist et al., 2018). It has been proposed that not only actions are under the influence of Pavlovian inhibitory mechanisms, but also our thoughts (Huys et al., 2012; Mendelsohn et al., 2014). Indeed, Huys et al. (2012); and Lally et al. (2017) recently provided empirical evidence that Pavlovian inhibitory processes have a central place in planning action sequences. This warrants future studies on the Pavlovian inhibitory mechanisms in especially impulsivity/hyperactivity in ADHD and with respect to MBCT that might advance our understanding of the neurocognitive mechanisms of both ADHD and the workings of MBCT, respectively.

One question that follows from the current study is why ADHD patients with clinically diagnosed impulsivity/hyperactivity lack the aversive Pavlovian inhibition we normally observe in healthy populations (Huys et al., 2011; Geurts et al., 2013a) and in ADHD patients without overt impulsivity/hyperactivity (this study). First, we note that instrumental and Pavlovian stimulus-outcome contingencies were learned by these patients as well as by the non-impulsive/hyperactive patients: Performance on query trials during conditioning nor (changes in) VAS-ratings of the Pavlovian stimuli across Pavlovian conditioning nor instrumental performance differed between these patient groups. Thus, the difference in aversive PIT cannot be readily explained by differences in learning. Moreover, it is not likely that within the PIT stage, these aversive Pavlovian CS were simply not noticed, because Pavlovian CS with appetitive valence exerted their normal (invigorating) effect (Huys et al., 2011). Thus, the absence of the inhibitory effect has to be searched downstream, in the interaction effect of Pavlovian and instrumental information itself. On a cognitive-psychological level this interaction effect might only surface when the aversive predictions are processed *and* used as guidance for steering instrumental behavior. Disturbances might thus come about through not processing the aversive information *as relevant* for behavioral procedures. The finding that patients showed increased effects of aversive Pavlovian stimuli post MBCT might be informative from this perspective. First, we note that the finding that MBCT increases the effect of aversive Pavlovian CS is in general accordance with a recent report on aversive Pavlovian conditioning (i.e., fear conditioning) before and after Mindfulness Based Stress Reduction (MBSR)(Hölzel et al., 2016). This study showed that through MBSR, healthy controls remained sensitive, as revealed by psychophysiological responses to the aversive Pavlovian CS (predictive of electrical shocks), whereas participants in the waitlist group lost this sensitivity. Our finding extends this result by showing that MBCT might potentiate the inhibitory effect of an aversive Pavlovian CS in adult ADHD patients. We speculate that this might be due to more openness to *guiding information* of contexts predicting adversity, instead of avoiding aversive information, in combination with an enhanced tendency to not immediately react, facilitated by the training. Moreover, on a neurophysiological level it has been shown that aversive Pavlovian inhibition depends on serotonergic signaling (Crockett et al., 2009; Geurts et al., 2013b; den Ouden et al., 2015) and is also influenced by methylphenidate suggesting catecholaminergic involvement (Swart et al., 2017). On a speculative account, we hypothesize that aberrant monoaminergic signaling related to Pavlovian control might be at the roots of this disinhibition, paralleling the psychological process by which aversive information guides instrumental behavior. Moreover, our data suggest that this process can be changed by MBCT.

Several limitations of this study should be noted: First we note that our relatively small sample size precluded us from assessing differential aspects of MBCT on the patients with the combined vs. the inattentive subtype of ADHD. This could have strengthened (or disproved) the suggestion that MBCT specifically remedies maladaptive aversive disinhibition. Moreover, including another active control treatment could have substantiated suggestions about the specificity of our result with regards to MBCT. With regard to the PIT paradigm, we think this might be improved by using a more naturalistic cover story (subjects informally reported that the game was boring) and more salient reinforcers (e.g., food, taste, shock, noise), which might make the task more ecologically valid and putatively more sensitive to change. Adding eye-tracking to this paradigm might also help to establish attentional components of the uncovered effect (e.g., more dwelling at the Pavlovian CS then at the instrumental stimulus) which might help to better understand the interindividual differences found here. Finally, because this paradigm has been shown to be sensitive to catecholaminergic modulation by methylphenidate and the current study suggests that it is also sensitive to change due to MBCT, it is interesting for future studies to assess whether this paradigm could have differential predictive properties in terms of treatment response for both pharmacological as well as psychotherapeutic interventions in ADHD.

In sum, our data suggests that the combined, but not the inattentive subtype of ADHD is associated with diminished aversive Pavlovian inhibition and that MBCT can enhance this inhibition. These findings offer new insights in the neurocognitive mechanisms of hyperactivity/impulsivity in the combined subtype of ADHD and point toward MBCT as an intervention that might influence these mechanisms.

## Data availability statement

The raw data supporting the conclusions of this article will be made available by the authors, without undue reservation.

## Ethics statement

The studies involving human participants were reviewed and approved by the METC Oost-Nederland. The patients/participants provided their written informed consent to participate in this study.

## Author contributions

DG, HO, RC, and AS contributed to conception and design of the study. DG, LJ, JS, and RC collected the data. DG performed the statistical analysis and wrote the first draft of the manuscript. All authors contributed to manuscript revision, read, and approved the submitted version.
