# Supplementary material for: Aversive Pavlovian inhibition in adult attention-deficit/hyperactivity disorder and its restoration by mindfulness-based cognitive therapy
Source: Front Behav Neurosci. 2022 Jul 25;16:938082. doi: 10.3389/fnbeh.2022.938082 (PMC9359138; doi:10.3389/fnbeh.2022.938082)
Supplement: Supplementary file 1 [file Data_Sheet_1.docx]

Supplementary Material

# Instrumental and Pavlovian conditioning

## Analyses

### Instrumental conditioning

There were two instrumental trial types, consisting of stimuli for which the correct response was either (i) collect (i.e. go) (ii) not collect (i.e. nogo). To assess instrumental conditioning, we calculated accuracy as the proportion of correct responses (p(correct)) over time (i.e. 2 time-bins for the first and last 10 trials of each instrumental stimulus for the instrumental conditioning). To assess whether patients learned to make the correct choice during instrumental conditioning, we used an rmANOVA with Time (first/last trial bin), Correct Choice (go/nogo) Day and Treatment.

### Pavlovian conditioning

We assessed differences in performance on the query trials (Figure 1C) between groups and days to examine whether patients paid attention and whether the associations between stimulus and outcome were explicitly learned. In addition we assessed VAS-ratings of the Pavlovian CS by means of a rmANOVA including Treatment, Day, Time (pre-/post-experiment) and Pavlovian CS Appetive and Aversive (5 levels: S^P^_++_ /S^P^_+_ /S^P^_n_ /S^P^_-_ /S^P^_--_) as factors.

## Results

Analyses of the instrumental and Pavlovian conditioning phases indeed reveal that patients acquired instrumental and Pavlovian contingencies as expected (see below for statistics). Thus, no MBCT or group effects were found on instrumental accuracy (No interactions with or main effect of Group within the rmANOVA Treatment x Correct Choice x Day x Time (beginning/end instrumental conditioning): all F<0.7, p>0.39). Furthermore, patients learned to make the correct responses during the instrumental conditioning phase: Accuracy significantly increased over time, meaning that patients learned the instrumental associations between stimuli, actions and outcomes (main effect of time: F(1,48)=4.9, p=0.032).

Moreover, no MBCT or group effects were found in terms of performance on the Pavlovian query trials on which participant had to make a forced choice between two Pavlovian CS (overall mean 84% correct, SD=15 no interactions with or main effect of Group within the rmANOVA Group x Day: all F<0.5, p>.482). In line with this finding we also did not find such effects on subjective liking ratings of the Pavlovian CS (no interactions with or main effect of Group within the rmANOVA Group x Day x Time x Pavlovian CS Valence: all F<2.4, p>.05). Furthermore, Pavlovian conditioning changed subjective liking of the Pavlovian CS in the expected direction, across both days without any significant difference between the Days (VAS ratings: time x Pavlovian CS Valence: F(1,48)=12.0, p<.001, figure S1). Thus, taken together, these results indicate that our general task manipulations worked as expected: subjects learned the instrumental and Pavlovian values independent of MBCT condition and testing day.

**Figure S1** VAS ratings before and after Pavlovian conditioning. This graph depicts successful Pavlovian conditioning, where post (red line) compared to pre (blue line) conditioning appetive Pavlovian CS were liked better and aversive CS were liked less. Graph represents means of VAS scores (0 = very aversive, 0.5 = neutral, 1 = very appetitive). Error bars represent SEMs.

# Specifying the MBCT induced increase in Pavlovian inhibition

In an exploratory analysis, suggested in a helpful comment of a reviewer, we assessed whether this increase in inhibition was due to a general increase in NoGo-actions or whether it represents an increase in appropriate NoGo-actions (i.e. only in response to the instrumental no-go-stimuli). Therefore, we added Required Action as a factor to the main generalized mixed effects model, indicating which action (Go or NoGo) would be correct (i.e. mostly rewarded) for the stimulus presented. MBCT did not differentially increase aversive inhibition for different instrumental stimuli, but increased inhibition irrespective of Required Action.

## Supplementary Figures


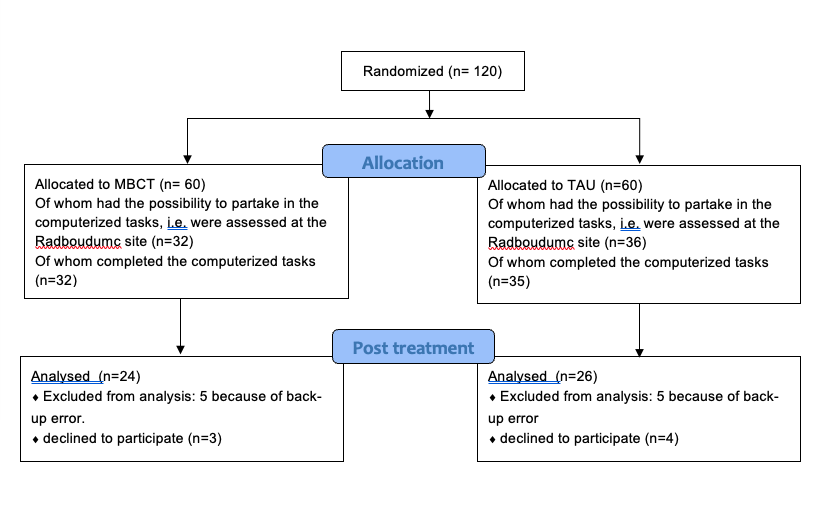


**Supplementary Figure 1.** Flow-chart of patient inclusion.
